# Neural Correlates underlying Size Constancy in Virtual Three-Dimensional Space

**DOI:** 10.1038/s41598-017-03652-6

**Published:** 2017-06-12

**Authors:** Jing Xia, Pengfei Wang, Qi Chen

**Affiliations:** 1 Center for Studies of Psychological Application and School of Psychology, South China Normal University, Guangzhou, 510631 China; 2 Guangdong Key Laboratory of Mental Health and Cognitive Science, South China Normal University, Guangzhou, 510631 China; 3 Center for Psychology and Brain Science and Department of Psychology, Guangzhou University, Guangzhou, 510006 China

## Abstract

The perceived size of an object remains relatively constant although its retinal size keeps decreasing as the object moves away along the depth dimension of the 3D space, i.e. size constancy. Neural mechanisms generating size constancy in virtual 3D space, however, remain poorly understood. By constructing a virtual 3D world in the MR scanner, we positioned the same 3D objects either near or far from the observers so that the near and far objects were perceived as having the same physical size despite their differences in retinal size. To control for the effect of differential retinal size, an additional 2D condition was introduced: a large and a small object, with matched retinal images as the near and far objects in the 3D condition, respectively, were presented on a 2D screen. Differences in retinal size activated overlapped areas in bilateral inferior occipital gyrus (IOG) in both experiments. The overlapped areas in IOG, however, showed different patterns of functional connectivity with different neural networks, depending on the perceived size of objects. In particular, IOG showed enhanced connectivity with bilateral superior parietal cortex in the 2D condition, but with inferior temporal and prefrontal cortex in the virtual 3D condition, i.e., size constancy.

## Introduction

Our perception of one particular object’s size remains relatively constant although its retinal size keeps decreasing as the object moves away from us along the depth dimension of the three-dimensional (3D) visual world. This phenomenon is termed as “size constancy”, which contributes to the perceived stability of the external visual world around us. It has been well documented that size constancy is based on the size-depth invariance principle, i.e. an approximately constant ratio between the perceived size of an object and its apparent distance in depth from the observer^1–4^.

Neural correlates underlying the size constancy effect have been investigated in both real^5^ and virtual 3D world^6^. For example, Sperandio *et al*.^5^ used the perceived size of an afterimage at different viewing distances in the MR scanner to investigate the neural mechanisms underlying size constancy in the real 3D world. Behaviorally, the afterimage of a small light was perceived as larger on a further rather than closer screen. At the neural level, neural activity in V1 was modulated by the perceived size of an afterimage even though the size of the retinal image remained the same. Moreover, by adopting the fMRI adaptation paradigm in a virtual 3D world, Weidner *et al*.^6^ investigated the neural correlates underlying size constancy effect in more detail. In their study, across different depth distances, both a condition with constant perceived size (variable retinal size) and a condition with variable perceived size (constant retinal size) were included. The fMRI results showed that neural activity in bilateral middle occipital gyrus was significantly adapted to trials in the variable perceived size (constant retinal size) condition, indicating the functional role of the middle occipital gyrus in recalculating the perceived size by conjointly taking into account both retinal size and depth distance. However, in the constant perceived size (variable retinal size) condition, where the classical size constancy effect occurs, no significant adaptation effect was reported. Therefore, it remains unclear how the lower level visual cortex interacts with higher-level cortical regions to explain the size-distance invariant object representations.

In addition to the neural mechanisms underlying the size constancy effect in real or virtual 3D world, many optical 2D size illusions induced by pictorial cues can also be explained by the principle of size constancy^7, 8^. For example, when two objects of the same physical size are presented in either a closer or further apparent depth on a 2D pictorial background, the seemingly further object is perceived to be larger than the seemingly closer one^7, 9^. This phenomenon is termed as the Ponzo illusion. Since in the 3D world, a further object would have to be larger than a closer object for both to produce identical retinal images, people misperceive the seemingly further object as being larger than the seemingly closer object. At the neural level, neural activity in V1 is modulated by the perceived rather than the retinal size of the object: the more distant object, which appears larger, activates a larger area in V1 than a closer object, which appears smaller, although the two objects are of equal retinal size^10, 11^.

Although previous studies have provided strong evidence towards the involvement of the occipital cortex in both the 3D size constancy effect^5, 6^ and the 2D size illusions^9, 10^, it remains unknown how size constancy is induced by the interaction between the primary/secondary visual cortex and the higher order brain areas. In particular, it has been well established that the key organization property in V1 is retinotopic mapping^12–14^. Size perception, however, is a function of higher-order visual areas such as lateral occipital cortex (LOC), inferior temporal (IT) cortex and prefrontal cortex^15–17^. Therefore, the involvement of V1 activity in perceiving 3D size constancy and 2D size illusions might be due to the top-down modulation from higher-level areas^10^. In the present fMRI study, by constructing a virtual 3D world in the MR scanner via 3D goggles, we aimed to investigate whether and how the functional connectivity between the occipital cortex and the higher order brain regions change as a function of the constant perceived size in virtual 3D space vs. varying perceived size in 2D.

In the present virtual 3D condition, the same 3D object was positioned either close to or far away from the observers so that the further object was perceived as the same object as the closer one (of the same physical size) despite their differences in retinal size, i.e. size constancy. To further control the bottom-up stimuli difference induced by differences^9^ in retinal sizes, a 2D control condition was introduced. A small or large 2D object was presented on a 2D screen, and most importantly, the small and large 2D objects were matched, in terms of the retinal sizes/images, with the far and near objects in the virtual 3D condition, respectively. Therefore, in the 2D control condition, the perceived object size was dependent on the retinal size: the object with smaller retinal size was perceived as smaller than the object with larger retinal size. In addition, in order to show that size constancy contributes to the perceptual stability of the virtual 3D world, irrespective of the type of behavioral tasks performed, three types of task were adopted in both the 3D and the 2D conditions: allocentric judgment, egocentric judgment, and color discrimination task. To further confirm whether human subjects perceive the far objects as the same objects as the near objects in the present virtual 3D environment, we also conducted a psychophysics control experiment. We predicted that the differences in retinal sizes will evoke identical neural activations in the occipital areas between the 2D (small vs. large) and 3D (far vs. near) conditions since the bottom-up stimuli were matched in retinal sizes between the two conditions. With regard to the neural correlates underlying size constancy in the virtual 3D condition and the perception of differential object sizes in the 2D control condition, we expected to observe differential patterns of functional connectivity between the commonly activated occipital visual areas and higher-order cortical areas.

## Results

### The general linear model (GLM) analysis

**Figure 1:**
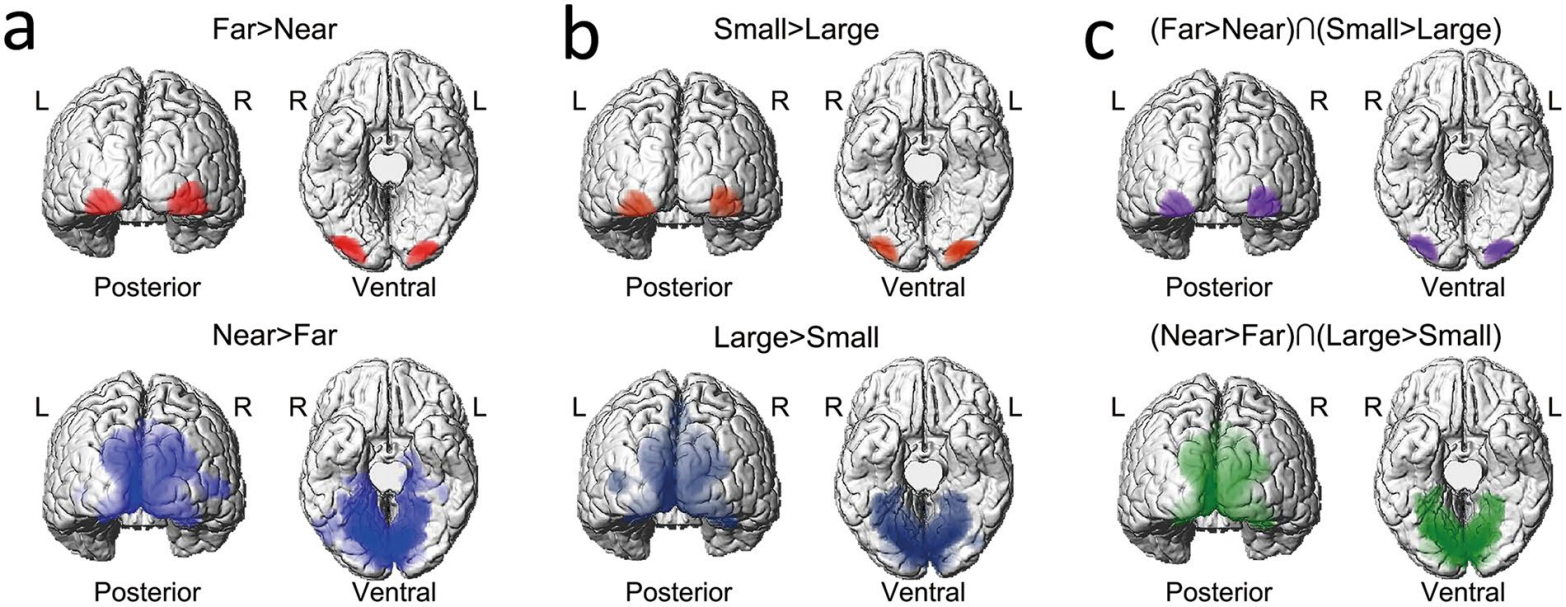
The main effect and conjunction result in the virtual 3D and 2D experiment. (**a**) Main effect of the depth of objects in the virtual 3D experiment. Red: “Far > Near”; Blue: “Near > Far”. (**b**) Main effect of the physical size of stimuli in the 2D experiment. Dark red: “Small > Large”; Dark blue: “Large > Small”. (**c**) The conjunction analysis between (**a**) and (**b**).

In the virtual 3D condition, bilateral inferior occipital gyrus (IOG) was significantly activated in the “Far” condition as compared to the “Near” condition (Fig. 1a, upper panel and Table 1a). On the other hand, bilateral superior occipital gyrus (SOG), extending to bilateral calcarine and bilateral lingual gyrus, was activated in the reverse contrast “Near > Far” (Fig. 1a lower panel and Table 1b).

**Table 1:** The main effect of the depth of objects in the virtual 3D experiment and the main effect of the size of stimuli in the 2D experiment.

Anatomical Region	Cluster Peak (mm)	*t*-Score	*k*E (voxels)
**The 3D experiment**			
**a. Far** > **Near**			
Right inferior occipital gyrus	32, −88, −10	6.30	643
Left inferior occipital gyrus	−32, −92, −10	5.44	453
**b. Near** > **Far**			
Left superior occipital gyrus	−10, −92, 8	10.91	17162
*Right lingual*	*18*, *−74*, *−6*	*10*.*77*	
*Left calcarine*	*−6*, *−88*, *2*	*10*.*34*	
*Left lingual*	*−6*, *−76*, *−4*	*9*.*44*	
*Right calcarine*	*14*, *−82*, *4*	*9*.*37*	
*Right superior occipital gyrus*	*26*, *−84*, *26*	*8*.*55*	
**The 2D experiment**			
**c. Small** > **Large**			
Left inferior occipital gyrus	−32, −96, −10	10.36	515
Right inferior occipital gyrus	34, −94, −4	8.70	474
**d. Large** > **Small**			
Left superior occipital gyrus	−10, −96, 12	10.53	12212
*Right lingual*	*16*, *−80*, *−10*	*8*.*65*	
*Right superior occipital gyrus*	*14*, *−94*, *18*	*8*.*63*	
*Right calcarine*	*18*, *−86*, *12*	*8*.*47*	
*Left calcarine*	*−6*, *−88*, *0*	*7*.*86*	
*Left lingual*	*−10*, *−86*, *−10*	*7*.*64*	
**e. (Far** > **Near) ∩ (Small** > **Large)**			
Right inferior occipital gyrus	32, −88, −10	6.30	452
Left inferior occipital gyrus	−32, −92, −10	5.44	423
**f. (Near** > **Far) ∩ (Large** > **Small)**			
Left superior occipital gyrus	−10, −94, 10	10.27	10011
*Right lingual*	*22*, *−72*, *−8*	*8*.*73*	
*Right calcarine*	*19*, *−84*, *10*	*8*.*06*	
*Left lingual*	*−20*, *−78*, *−14*	*7*.*88*	
*Left calcarine*	*−6*, *−88*, *0*	*7*.*86*	
*Right superior occipital gyrus*	*20*, *−94*, *28*	*5*.*86*	

The coordinates (x, y, z) correspond to MNI coordinates. Displayed are the coordinates of the maximally activated voxel within a significant cluster as well as the coordinates of relevant local maxima within the cluster (in Italics).

Similarly, in the 2D control condition, bilateral IOG was significantly activated in the “Small” condition as compared to the “Large” condition (Fig. 1b upper panel and Table 1c). On the other hand, bilateral SOG, extending to bilateral calcarine and bilateral lingual gyrus, was activated in the reverse contrast “Large > Small” (Fig. 1b lower panel and Table 1d).

The neural network involved in the main effect of the depth of objects in the virtual 3D condition mostly overlapped with the neural network involved in the main effect of the physical size of stimuli in the 2D condition. To isolate the common neural correlates underlying the two main effects, we further performed a conjunction analysis between them. The results showed significant activations in bilateral IOG in the “(Far > Near) ∩ (Small > Large)” conjunction contrast (Fig. 1c upper panel and Table 1e). On the other hand, bilateral SOG, extending to bilateral calcarine and bilateral lingual gyrus, was significantly activated in the reverse conjunction contrast, i.e., “(Near > Far) ∩ (Large > Small)” (Fig. 1c lower panel and Table 1f).

### Psychophysiological interaction (PPI) analysis with bilateral inferior occipital gyrus as the source regions

**Figure 2:**
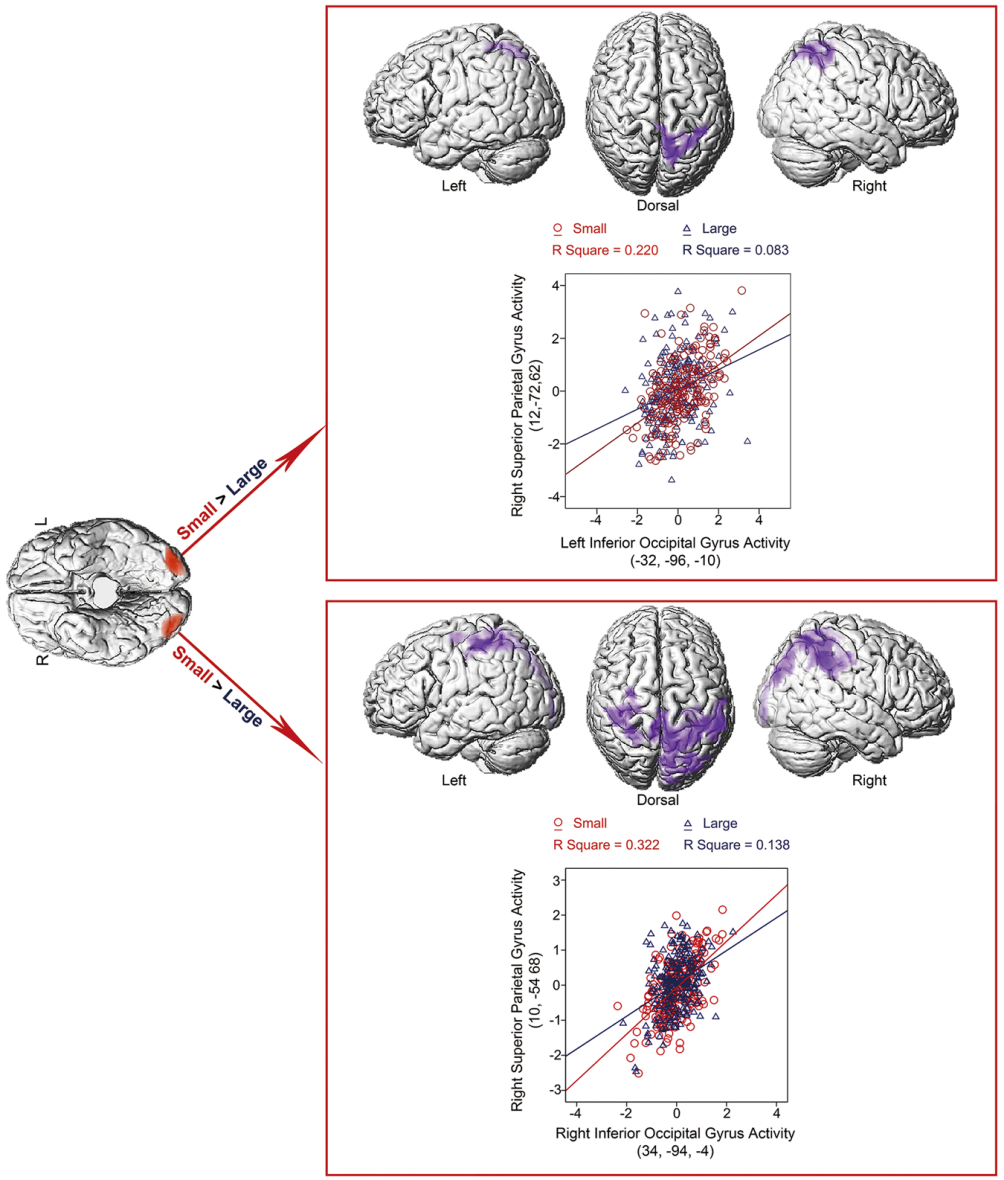
PPI analysis based on neural activity in bilateral IOG (dark red) with the “Small > Large” contrast as the psychological factor in the 2D experiment. The right upper panel: the left IOG showed higher neural coupling with the right SPC in the “Small” condition than in the “Large” condition. PPI analysis based on neural activity in the left middle occipital gyrus of an individual subject (Subject 2D_S24) is shown. Mean corrected neural activity in the right SPC was plotted as a function of the mean corrected neural activity in the left IOG in the “Small” and “Large” conditions, respectively. The right IOG showed higher neural coupling with bilateral SPC in the “Small” condition than in the “Large” condition. PPI analysis based on neural activity in the right IOG (Subject 2D_S24) was shown. Mean corrected neural activity in bilateral SPC was plotted as a function of the mean corrected neural activity in the left IOG in the “Small” and “Large” conditions, respectively.

In the 2D control condition, bilateral IOG showed higher neural coupling with the right superior parietal lobule in the “Small” condition than in the “Large” condition (Fig. 2 and Table 2a).

**Table 2:** Results of the PPI analyses in the 2D and virtual 3D experiment.

Anatomical Region	Cluster Peak (mm)	*t*-Score	*k*E (voxels)
**a. The 2D experiment**			
**Left inferior occipital as seed, with “Small** > **Large” as the psychological factor**			
Right superior parietal gyrus	12, −72, 62	4.51	742
**Right inferior occipital as seed, with “Small** > **Large” as the psychological factor**			
Right superior parietal gyrus	10, −54, 68	5.36	4230
*Left superior parietal gyrus*	*−12*, *−48*, *70*	*4*.*64*	
**b. The 3D experiment**			
**Left inferior occipital as seed, with “Far** > **Near” as the psychological factor**			
Right inferior frontal gyrus	30, 40, −16	7.77	704
Right inferior temporal gyrus	66, −42, −8	5.83	1139
Right superior frontal gyrus	10, 36, 52	5.24	527
**Right inferior occipital as seed, with “Far **>** Near” as the psychological factor**			
Right inferior frontal gyrus	52, 38, 2	3.90	2499
*Right superior frontal gyrus*	*10*, *40*, *52*	*3*.*78*	
Right inferior temporal gyrus	58, −56, −4	3.71	921

The coordinates (x, y, z) correspond to MNI coordinates. Displayed are the coordinates of the maximally activated voxel within a significant cluster as well as the coordinates of relevant local maxima within the cluster (in Italics).

**Figure 3:**
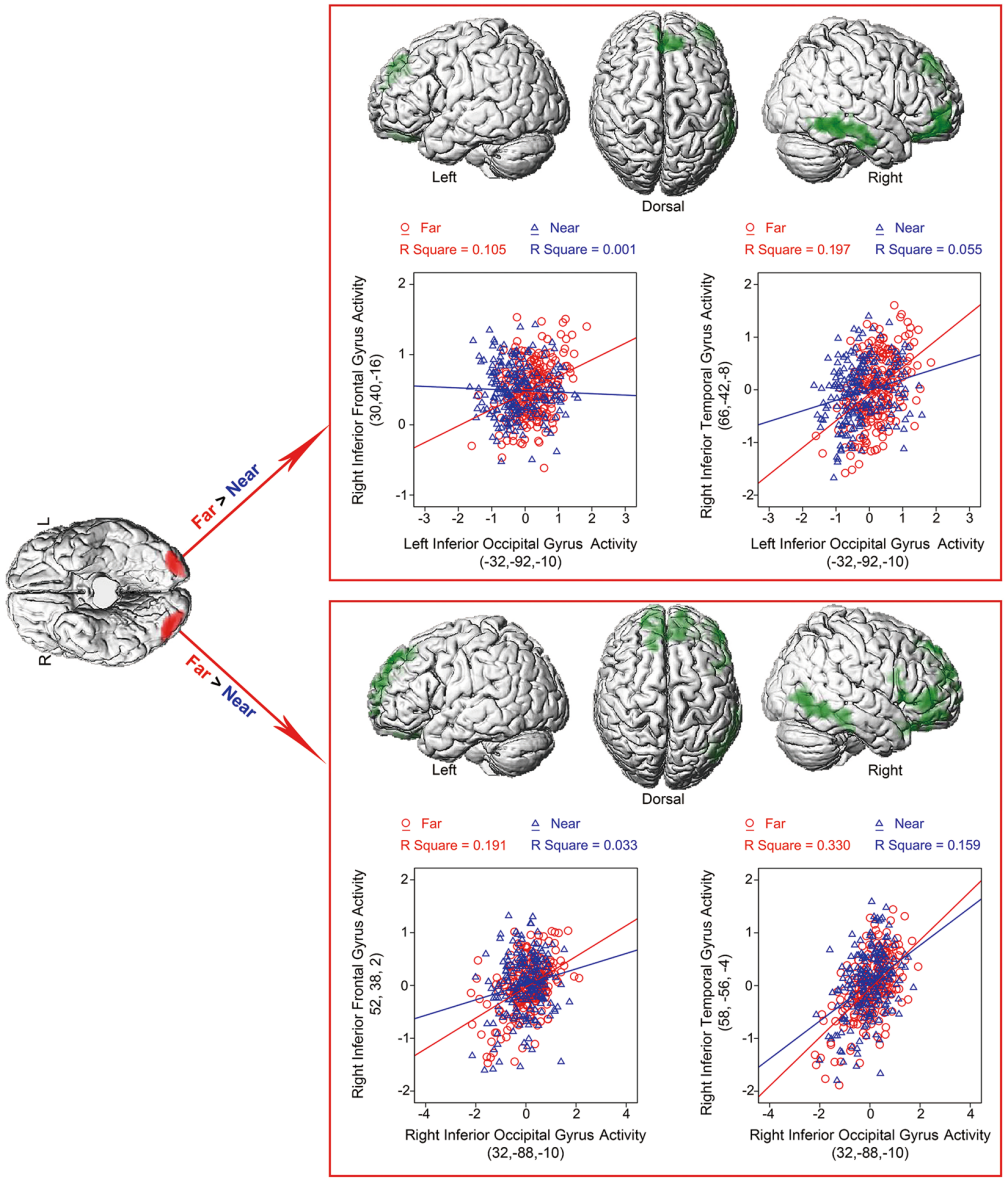
PPI analysis based on neural activity in bilateral IOG (red) with the “Far > Near” contrast as the psychological factor in the virtual 3D experiment. The right upper panel: the left IOG showed higher neural coupling with the right IFG, the right SFG and the right ITG in the “Far” condition than in the “Near” condition. PPI analysis based on neural activity in the left IOG (Subject 3D_S13) was shown. Mean corrected neural activity in the right IFG and the right ITG was plotted as a function of the mean corrected activity in the left IOG in the “Far” and “Near” conditions, respectively. The right lower panel: the right IOG showed higher neural coupling with the right IFG, the right SFG and the right ITG in the “Far” condition than in the “Near” condition. PPI analysis based on neural activity in the right IOG (Subject 3D_S13) was shown. Mean corrected neural activity in the right IFG and the right ITG was plotted as a function of the mean corrected activity in the right IOG in the “Far” and “Near” conditions, respectively.

In the virtual 3D condition, with the “Far > Near” contrast as the psychological factor, PPI analysis based on the neural activity of identical seed areas showed a different pattern from the 2D condition. The left IOG showed higher neural coupling with the right inferior frontal gyrus, the right superior frontal gyrus and the right inferior temporal gyrus in the far than near condition. The right IOG showed higher neural coupling with the right inferior frontal gyrus, the right superior frontal gyrus and the right inferior temporal gyrus in the far than near condition (Fig. 3 and Table 2b), which failed to pass the multiple comparisons correction at the cluster level (voxel threshold at *p* < 0.05, cluster size more than 800 voxels).

We also used the SOG (derived from the conjunction analysis between “3D_Near > 3_Far” and “2D_Large > 2D_Small”, Fig. 1c, lower panel) as the seeds to perform the PPI analysis. No significant PPI was found between SOG and other brain areas in either the 2D or the 3D condition.

### The behavioral data

**Figure 4:**
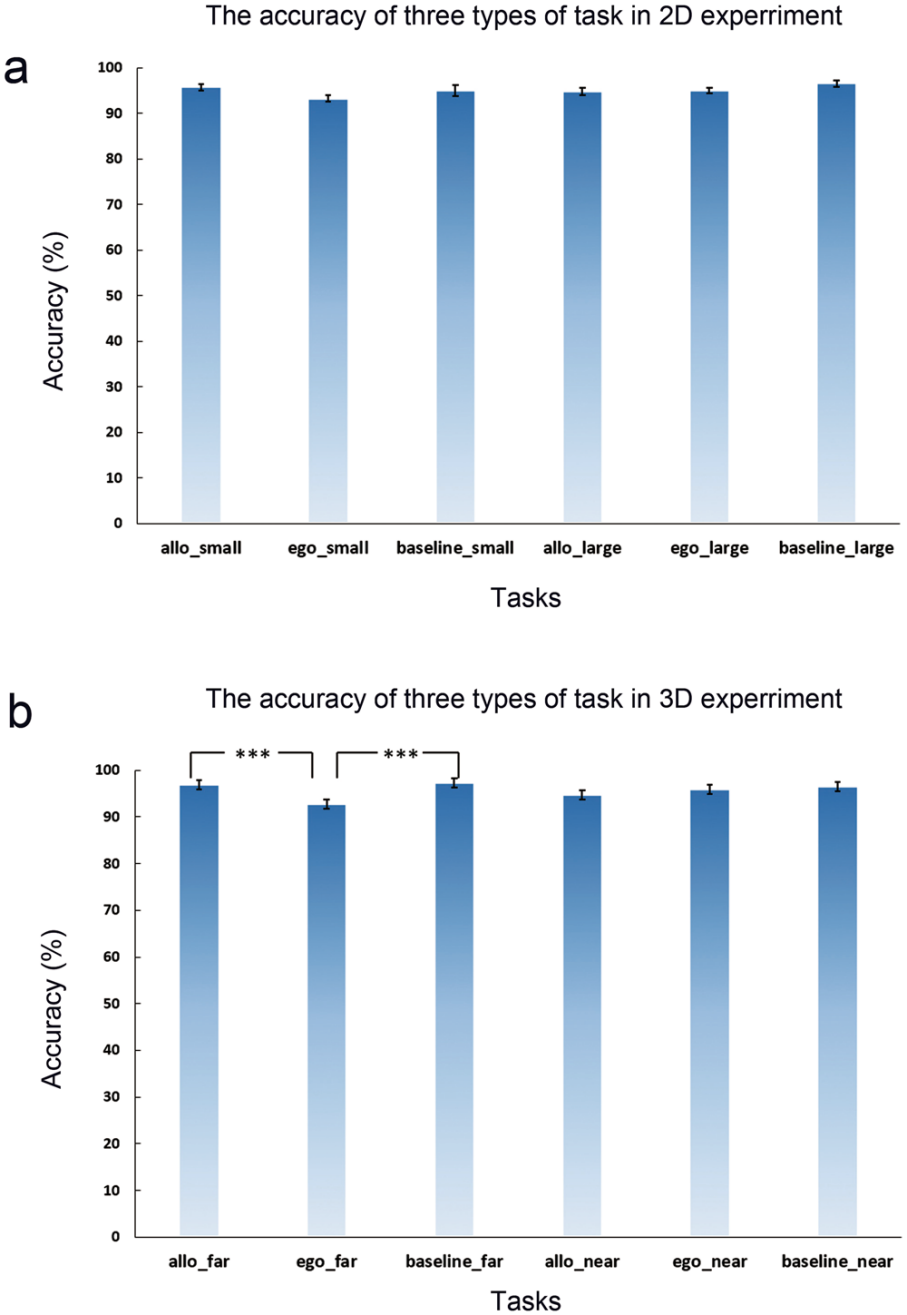
The accuracy of three types of task in 2D and 3D experiment. (**a**) The accuracy of three types of task in 2D experiment. (**b**) The accuracy of three types of task in 3D experiment.

Behaviorally, accuracy of the three types of task was submitted to a 2 (near vs. far in 3D; small vs. large in 2D) × 3 (type of tasks: allocentric judgment, egocentric judgment, and color discrimination) repeated-measures ANOVA for the 2D and the 3D experiment, respectively. For the 2D experiment, neither the two main effects nor the interaction was significant, all *p* values > 0.05 (Fig. 4a), indicating that participants performed the three types of task equally well for both the small and the large objects. For the 3D experiment, both the main effect of the three types of task, *F*_(2, 36)_ = 9.618, *p* < 0.001, and the interaction, *F*_(2, 36)_ = 9.996, *p* < 0.001, was significant. The main effect of depth (near vs. far) was not significant, *F* < 1. Further planned pair-wise comparisons (with Bonferroni correction) on simple effects showed that accuracy was significantly lower in the egocentric than the allocentric, *p* = 0.002, and the color discrimination, *p* < 0.001, tasks, only in the far depth plane, but not in the near depth plane (Fig. 4b). These results indicated that although the far objects were perceived as the same objects as the near objects in the 3D condition, egocentric judgments were more difficult on far than near objects.

In the present study, we hypothesized that the early perceptual processing (i.e. perception of size constancy in the 3D world and perception of size difference in the 2D world) should be constant across the three types of experimental task within the 3D_Near, the 3D_Far, the 2D_Large, and the 2D_Small conditions. Moreover, since neither the main effect of far vs. near objects in the 3D condition nor the main effect of small vs. large objects in the 2D condition was significant, we were able to localize the differential neural effect between the early perceptual processing of the far vs. near objects in the 3D condition and between the small vs. large objects in the 2D condition, irrespective of the type of behavioral tasks, by collapsing over the three types of task.

### The psychophysics control experiment

In the natural environment, for example, if a car drives away from us, we perceive the distant car as the same object as when it is close to us, without the needs to test its perceived size. This is why we directly put the same objects to either near or far space in the virtual 3D condition of the present study. However, there exist differences between depth perception in the real and the virtual 3D environment. For example, compared to the real 3D world, distances from the observer were underestimated in the virtual environments^18^. Furthermore, distance miscalculation was universal in various virtual reality devices^19–21^. Therefore, to further study whether human subjects perceive the far objects as the same objects as the near objects in the present virtual 3D environment, we conducted another psychophysics control experiment. A new group of participants was asked to adjust the size of the far objects until they reported that the far objects were perceived to be the same objects as the near objects. We predicted that if size constancy existed in the present virtual 3D environment, there should be no significant difference between the size of the far object in the formal experiment, and the subjectively adjusted size of the far object in the psychophysics control experiment. The size constancy effect should exist irrespective of whether the near objects were directly put in far space in the formal experiment or the participants subjectively adjusted the size of the far objects until they perceived the far objects as the same as the near objects in the psychophysics control experiment.

The average adjusted size of the far plate, at which the participants perceived the far objects as the same objects as the near objects, was submitted to a one sample *t* test to compare with the size of the far plate when the near objects were directly put in the far space of the virtual 3D environment. The results suggested that there was no statistically significant difference between the modulated size (7.77° ± 0.19 in visual angle) and the real size of the far plate (7.67° in visual angle), *t* (8) = 0.345, *p* > 0.05, Cohen’s *d* = 0.244. These results thus indicated that participants perceived the 3D_Far object as the same object as the 3D_Near object in the 3D virtual condition, irrespective of whether the participants were asked to subjectively adjust the size of the far objects or whether near objects were directly put in the far space.

## Discussion

In the present fMRI study, we aimed to investigate how size constancy was generated in the virtual 3D condition while the perception of small vs. large objects was generated in the 2D condition based on the same difference in the retinal sizes, with emphasis on the functional connectivity between the low level occipital areas and the higher level cortical regions. In the virtual 3D condition, participants perceived the further object as having the same physical size as the closer object despite their differential retinal sizes (i.e., size constancy). In the 2D control condition, we removed the depth dimension while keeping the difference in retinal size the same as that in the virtual 3D condition so that participants perceived the object with smaller retinal size as physically smaller than the object with larger retinal size. It has been suggested that familiar size based on past experience is a cue to perceived depth: larger sizes imply closer distance while smaller sizes imply further distance^22^. In the present 2D experiment, however, there was neither binocular disparity nor pictorial cues being presented on the 2D screen, and participants were explicitely informed that objects of different sizes would be presented on the same 2D screen with the same viewing distance. We, therefore, do not think there were significant differences in the perceived depth between the small and large stimuli in the 2D experiment.

**Figure 5:**
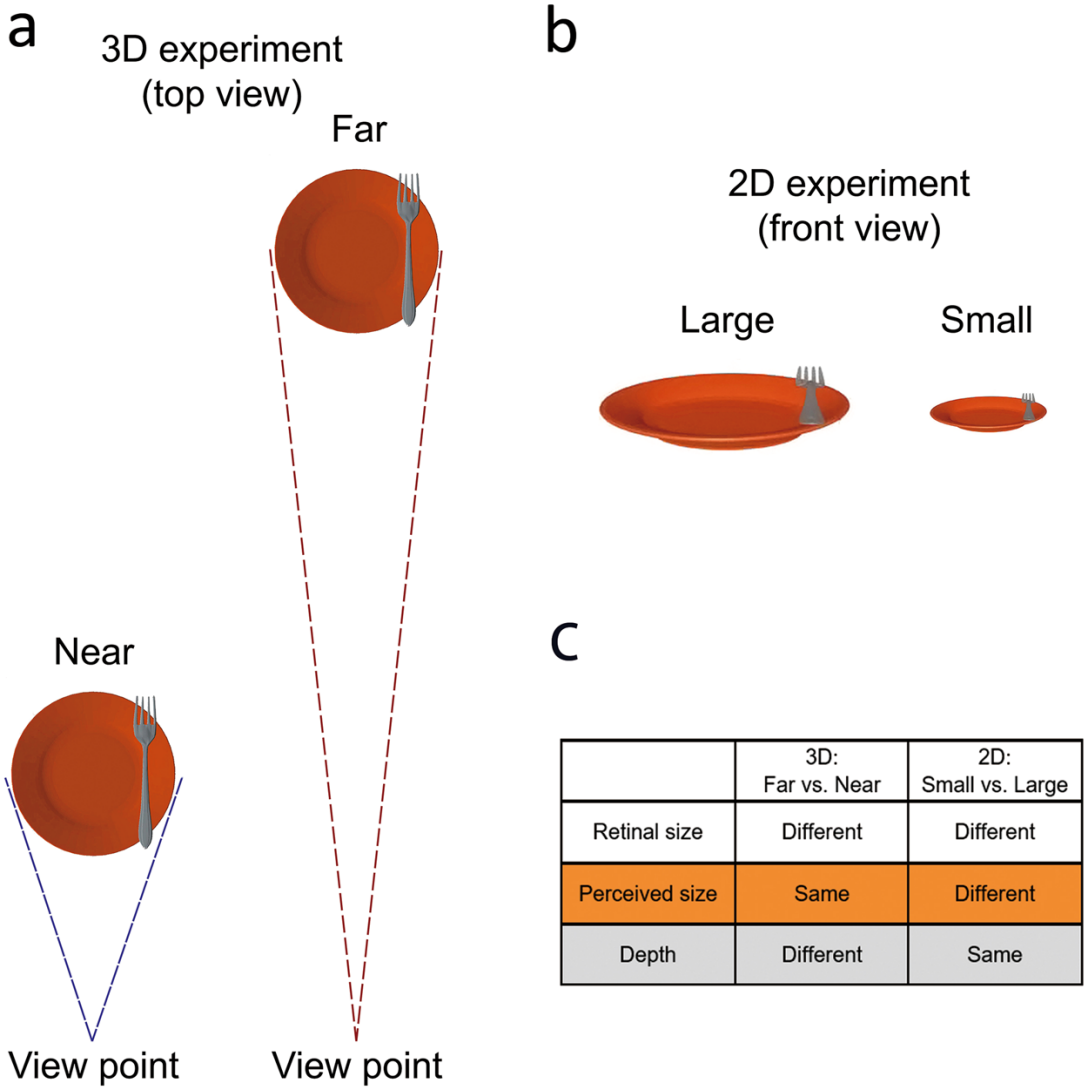
Experimental settings in the present study. (**a**) Top view of the 3D objects in the virtual 3D experiment. (**b**) Visual stimuli in the 2D experiment were exactly the same as the frontal view of the 3D stimuli in the virtual 3D experiment. (**c**) Two major differences (hightlighted) between the 3D and the 2D experiment.

Please note that there were two critical factors in the 3D condition, compared to the 2D condition: (1) the differential depth distance, and (2) the constant perceived size between the near and far objects (Fig. 5c). The different depth distance in the 3D world is a prerequisite condition that drives the constant perceived size of the same object at the near and far depth distance. Therefore, the two factors together constitute the experimental variant between the 3D and 2D condition, and cannot be dissociated. Specifically speaking, in the present virtual 3D condition, there was perceived depth difference between the 3D_Near and 3D_Far conditions, which was driven by binocular disparity. In contrast, no such difference was present between the 2D_Large and 2D_Small conditions in the 2D control condition, in which size constancy accordingly did not exist. What was kept constant between the 3D and the 2D conditions was the difference in retinal size between the 3D_Near and the 3D_Far stimuli and between the 2D_Large and the 2D_Small stimuli (Fig. 5c). Therefore, the purpose of the present design was to investigate, with the same difference in retinal inputs between the 3D_Near and 3D_Far stimuli, and between the 2D_Large and the 2D_Near stimuli, why size constancy was generated in the 3D condition, but the perception of different sizes of the object was generated in the 2D condition. In particular, we were interested in how the same occipital activations, driven by the same difference in bottom-up retinal inputs, interact with different higher order neural regions to generate the perception of size constancy in the 3D condition and the perception of differential sizes in the 2D condition.

At the neural level, the comparison between the “Far” and “Near” stimuli in the virtual 3D condition activated identical occipital regions as the comparison between the “Small” and “Large” stimuli in the 2D condition: the smaller and the further stimuli commonly activated ventral bilateral IOG; the larger and the closer stimuli commonly activated bilateral SOG (Fig. 1 and Table 1). Since the bottom-up stimuli in the 2D and 3D conditions were matched in retinal size, the above differential neural activations in the occipital cortex were evoked by differences in retinal size of the stimuli. The present results are consistent with previous evidence from a PET study, in which neural activity elicited by physically large and small 2D figures were compared, and similar differential activations in the occipital cortex were found as those in the present 2D condition^23^. Please note that all the stimuli in Fink *et al*.^23^ were always presented at the center of the screen, while the stimuli in the present study were presented in one of the four horizontally arranged positions, with the restriction that the number of trials at each of the four positions was equal. Therefore, the present results, together with previous evidence, suggested that differential neural activations in bilateral ventral IOG and in bilateral SOG reflected the smaller and the larger retinal sizes, respectively, irrespective of the spatial location of the bottom-up stimuli.

Previous evidence on 2D optical illusions induced by pictorial depth cues, such as the Ponzo illusion, suggested that neural activity in V1 was modulated by the perceived rather than the retinal size of objects: even with the same retinal size, the illusorily perceived larger object activated more extended areas in V1 than the perceived smaller object^10, 11^. Moreover, previous evidence on neural correlates underlying the size constancy effect in real 3D world also indicated that neural activity in V1 was modulated by the perceived size of an afterimage even though the size of the retinal image remained the same^5^. In the virtual 3D condition of the present study, the closer stimuli in the near blocks were directly positioned further away in the far blocks. Therefore, the closer objects were perceived as having the same physical size as the further stimuli due to size constancy although they differed in retinal sizes. Bilateral lingual gyrus in V1 showed higher neural activity to the closer stimuli than the further stimuli (Fig. 1a upper panel). Since the closer and the further objects had the same perceived size and differed in retinal size, the differential neural activity in V1 was caused by the retinal size, rather than the perceived size, the larger the retinal size, the higher the neural activity. Therefore, it seemed that the present results came to a conclusion opposite to the conclusion from previous studies on 2D illusions and real world size constancy. Please note, the retinal size was kept constant and the perceived size varied in the previous 2D illusion studies. In the present virtual 3D experiment, however, the perceived size was kept constant and the retinal size varied. The present results reflected the topographical property in V1, which has been well documented in the previous studies^12–14, 24^. By contrast, in the previous 2D illusion studies^10^, differences in the retinal topographical organizations between the apparent further and closer objects were eliminated (i.e., the two objects were of equal retinal size). Therefore, any difference in V1 evoked by the two objects should be mainly driven by the top-down modulation from higher level areas. It has been accordingly suggested that when the retinal size of objects was the same, the top-down modulations from higher-order cortical regions altered neural activity in V1^10, 15–17, 24^. In addition to the converging evidence on V1 both during 2D illusions^10, 11^ and in real 3D world^5^, it has been suggested that bilateral middle occipital gyrus is also implicated in the condition of variable perceived size (constant retinal size) in the virtual 3D world^6^. However, the neural mechanisms underlying the size constancy effect in the condition of constant perceived size (variable retinal size) in the 3D world remains unclear.

Therefore, a critical question to be answered is how, based on the same differences in bottom-up retinal inputs, size constancy is generated between the near and far objects in the virtual 3D space while the sense of different object size is generated on the 2D screen. In order to answer this question, we performed PPI analysis using the same bilateral IOG areas, which showed enhanced neural activity to the further objects in the 3D condition and to the smaller objects in the 2D condition, as the source regions in the virtual 3D and the 2D condition, respectively. In the 2D condition, bilateral IOG showed significantly enhanced functional connectivity with the superior parietal cortex (SPC) in the “Small” condition as compared to the “Large” condition (Fig. 2 and Table 2a). Therefore, the enhanced neural coupling between bilateral IOG and the SPC may contribute to the size perception of physically smaller objects with smaller retinal sizes. The pattern of connectivity in the 2D condition fitted well with evidence from previous patient studies. For instance, although one patient with parietal lesions was able to understand functions of the visual features of objects, they always under-estimate the size of the objects^25^. In addition, children with a perceptual disorder involving brief episodes of visual distortions (i.e. the Alice in Wonderland syndrome) showed increased neural activation in the SPC^26^.

In the virtual 3D condition, the same occipital regions in bilateral IOG, however, showed significantly enhanced functional connectivity with the inferior frontal gyrus (IFG), the superior frontal gyrus (SFG) and the inferior temporal gyrus (ITG) in the “Far” condition as compared to the “Near” condition (Fig. 3 and Table 2b). Therefore, when an object moved further away from the observer along the depth dimension in virtual 3D space, the enhanced neural coupling between bilateral IOG and the prefrontal-temporal regions is involved in generating size constancy to keep a stable size perception of objects. A large body of brain imaging literature points to a particular functional role of the ITG in size perception. The ITG is considered to be the final station of the ventral visual processing pathway and is well suited to process and represent information about complex visual objects^27–29^. More importantly, the ITG has object-selective responses that are tolerant to changes in retinal size, position, and viewpoint - a hallmark of high-level object representations^15, 17, 30–34^. In terms of the phenomenon of size constancy, the same object in the near and far depth plane is identified as the same object with constant object size, irrespective of differences both in retinal size and depth distance. In the present study, bilateral inferior occipital gyrus (IOG) was significantly activated by the stimuli with smaller retinal size (2D_Small and 3D_Far stimuli), compared to the stimuli with larger retinal size (2D_Large and 3D_Near stimuli) (Fig. 1c, upper row). Although bilateral IOG was generally responsive to the bottom-up stimuli with smaller retinal sizes, it showed differential patterns of functional connectivity with different cortical regions, depending on whether the objects with smaller retinal sizes were perceived as smaller (in the 2D condition) or perceived as the same objects with the same size as the closer objects (in the 3D condition). Particularly in the 3D condition, when the far stimuli with smaller retinal size were perceived as the same objects with the same size as the near stimuli with larger retinal size, bilateral IOG showed enhanced functional connectivity with the ITG (Fig. 3). The enhanced connectivity between the IOG and the ITG during the size constancy effect implied that size-varying retinal representation in the IOG may be transformed into the size-invariant object representations in the ITG during the 3D condition, which is consistent with the functional roles of ITG in code objects irrespective of retinal size, position and viewpoint etc. Another important component of the neural circuit involved in object perception is the IFG. The IFG often shows larger activations for object images than for non-object images^35–37^. The IFG also shows functional roles in object encoding, manipulation (e.g., mental rotation, the perception of different views) and memory retrievals^17, 36, 38^. More importantly, the IFG plays a crucial role in top-down modulations^17, 39^. Therefore, the increased neural coupling between IOG, ventral visual stream and prefrontal cortex with the further depth in virtual 3D space might reflect the top-down modulation from higher-order cortical regions to keep the stable perception of the virtual 3D world.

Our visual experience is a combination of bottom-up processing of retinal inputs and top-down processing driven by higher-order brain areas. Evidence from 2D optical illusion studies, brain lesion studies and neuroimaging studies highlighted the primary visual cortex as an important node in mediating size constancy^10, 11, 40–43^. The effects of distance on size perception (i.e., size constancy) are well illustrated by optical illusions^44^. An influential Bayesian account was proposed to explain size constancy^45^. In this model, our brain makes perceptual decisions by calculating a balanced combination between the received sensory information (e.g., retinal images) and knowledge acquired from prior experience^46^. The rules, which the brain learns from previous experience, are essential for the perceptual decision. One critical rule is that one object’s physical property (e.g., size, shape and color) always keeps constant regardless of changes in retinal information that arises from changes in viewing distance, the angle of perspective, and lighting conditions. This is also the basic rule underlying size constancy. A similar theory on the mechanisms of size constancy is the theory of inappropriate constancy scaling^47, 48^. This theory proposes that people experience optical illusions because the human brain tends to keep the size of objects constant. According to the logic of this theory, size constancy is adopted to counteract variations in retinal images which change with distance, so that the size of one object is perceived to be the same. In the present study, since the bottom-up stimuli in the 2D and virtual 3D conditions were matched in retinal size, the pattern of neural activations in the occipital cortex induced by retinal inputs was similar in the two conditions. In the virtual 3D condition, bilateral IOG showed significantly enhanced functional connectivity with the IFG, SFG and ITG in the Far condition compared to the Near condition (Fig. 3 and Table 2b). The higher-order brain areas in IFG, SFG and ITG are responsible for size perception, object encoding and manipulation of objects^15, 17, 30, 31, 36, 38^. Based on the Bayesian model and the inappropriate constancy scaling model, the higher-order brain regions, which showed enhanced functional connectivity with the bilateral IOG specifically in the virtual 3D condition, might be involved in adopting previous experience about one object to counteract variations in retinal images so that the object size was kept constant irrespective of the varying retinal images between the “3D_Far” and the “3D_Near” conditions.

Taken together, we showed in the present study how our brain generated size constancy in virtual 3D space based on identical retinal inputs as those in 2D space. With an object moving further away from us, enhanced functional connectivity between the early visual cortex, the higher-order ventral visual stream in the inferior temporal cortex, and the prefrontal cortex enables us to keep a stable perception of the object irrespective of changes in the depth and the retinal size.

## Materials and Methods

### 3D Experiment

#### Participants

Nineteen healthy volunteers (7 male and 12 female, 24 ± 3 years old) participated in the virtual 3D experiment. They were all right-handed and had normal or corrected to normal visual acuity. None of them had a history of neurological or psychiatric disorders. Written and informed consent was obtained from all subjects before participation according to the guidelines of South China Normal University’s Human Subjects Review Board. All experimental protocols were approved by the Academic Committee of School of Psychology, South China Normal University. All methods were carried out in accordance with approved guidelines and regulations. All the participants were paid for their participation.

#### Experimental design and stimuli

A goggle-based MR-compatible system (VisuaStim Digital, Resonance Technologies, Northridge, CA) provided two separate VGA with digital dual video inputs for stereoscopic display, each with a resolution of 800 (horizontal) ×600 (vertical) pixels at 60 Hz refresh rate. The horizontal extent of the field of view was 30°. The default viewing distance was 75 cm. This default distance value has nothing to do with perceived depth of the near and far objects in the 3D conditions. In the 3D_Near condition, the 3D stimuli popped out of the default screen; in the 3D_Far condition, the 3D stimuli appeared behind the default screen. The dual-display stereoscopic video, with 0.5 mega pixel resolution in a 0.25 square area, yielded 3D images by delivering slightly disparate images to each eye (binocular disparity).

The stimuli consisted of two virtual 3D objects: a fork on the top of a round orange plate (Fig. 5a). Fork and plate were chosen as the stimuli because they are familiar objects in our daily life. The virtual 3D objects were generated by Blender (free open source 3D content creation software, www.blender.org), exported as DirectX files, and presented on a gray background by custom-made Presentation scripts (Presentation Software package, Neurobehavioral Systems, Inc., Albany, CA). The diameter of the plate was 15° of visual angle, and the nearer end of the fork was 2.5° of visual angle. In each trial, these stimuli were presented either in near or far space. In the near space condition, the virtual 3D objects popped out of the default screen of the goggles, and in the far space condition, the objects appeared behind the default screen. The distance from the center of the plate to the participants’ eyes was 50 cm in the 3D_Near condition and the distance was 150 cm away from the participants’ eyes in the 3D_Far condition. The different target distances were simulated by adjusting binocular disparity. The close and far space stimuli were presented at the same height (y = 0, at the level of the eyes) and tilted by 13° toward the participants. No fixation was presented in the experiment. Stimuli were presented either to the left or right visual field. The fork was presented at one of four horizontally aligned egocentric positions with regard to the center of the visual field, with two positions on the left and another two positions in the right visual field. For each of the four egocentric locations of the fork (−5°, −3.5°, 3.5°, 5°), the location of the plate was varied independently around the fork, using four allocentric positions (−2.4°, −1.7°, 1.7°, 2.4°). The visual angles of the eccentricity of the spatial locations of the stimuli were matched between the near and far stimuli in the 3D condition, and between the large and small stimuli in the 2D condition. Therefore, the spatial positions of the stimuli in the visual field could not affect the current neural activations. For each of the four experimental conditions (3D_Near, 3D_Far, 2D_Large, 2D_Small), stimuli on different spatial locations were collapsed during data analysis. As for control for eye movements, participants were instructed to look straight forward without moving their eyes. The importance of not moving eyes was repeatedly emphasized. Compared with the near space condition, objects presented far from the observers occupied a smaller area in the retina but were perceived as the same size. All the participants reported, during the practice block prior to the formal test, that they perceived the far object as the same object as the near object. Therefore, observers always had size constancy in the entire virtual 3D experiment, conjointly considering the size of retinal image and variations in viewing distance.

A block fMRI design was adopted and there were two types of blocks: the near blocks in which all the stimuli were presented in near space; and the far blocks in which all the stimuli were presented in far space. There were 24 repetitions for each of the two types of block, resulting in 48 blocks in total which were randomly presented. Each block consisted of ten trials, and each trial lasted for 1650 ms, resulting in a total block duration of 16.5 s. The target presentation time during each trial was 150 ms, leaving 1500 ms for the participants to give responses. For the near (24 blocks) and far (24 blocks) conditions, participants were asked to perform three types of tasks, i.e., allocentric judgment (8 blocks), egocentric judgment (8 blocks), and color discrimination (8 blocks). In the allocentric judgment task, participants were asked to judge whether the fork was on the left or right side of the plate. In the egocentric judgment task, participants were asked to judge whether the fork was on the left or right side of the midsagittal line of their own body. In the color discrimination judgment task, participants were asked to discriminate whether the fork had a high or low luminance. These three types of tasks were collapsed during data analysis for each of the four experimental conditions since we hypothesized that size constancy existed irrespective of task demands. Error trials were defined as the trials in which participants either gave wrong responses compared to the assigned correct responses (incorrect trials) or did not give any responses (missed trials). Trials with RTs ± 3 SD away from the mean RT were defined as outlier trials and excluded from further analysis. The correctly responded outlier trials, however, were not counted as the error trials. The accuracy was calculated as the ratio between the correct trials (all trials except for error trials) and the total number of trials in each experimental condition of each participant. The accuracy data were submitted to a 2 (near vs. far in 3D; small vs. large in 2D) × 3 (type of tasks: allocentric judgment, egocentric judgment and color discrimination) repeated-measures ANOVA for the 2D and the 3D experiment, respectively. Participants performed the behavioral tasks equally well in the 2D and 3D experiments, with the mean accuracy higher than 95% in both tasks. The apparatus and experimental tasks used in this experiment were the same as those in a previous study (please see Chen *et al*.^49^ for more details). Since we assumed that the perception of far vs. near object (i.e., size constancy) in the virtual 3D experiment, and the perception of small vs. large object in the 2D experiment occurred irrespective of the behavioral tasks performed on the objects, we combined the three types of task blocks into the near and far blocks in the virtual 3D experiment, and the large and small blocks in the 2D experiment.

#### Data acquisition and pre-processing

A 3T Siemens Trio system with a standard head coil (Erlangen, Germany) was used to obtain T2*-weighted echo-planar images (EPI) with blood oxygenation level-dependent (BOLD) contrast (matrix size: 64 × 64, voxel size: 3.1 × 3.1 × 3.0 mm3). Thirty-six transversal slices of 3 mm thickness that covered the whole brain were acquired sequentially with a 0.3 mm gap (TR = 2.2 s, TE = 30 ms, FOV = 220 mm, flip angle = 90°). The one-run functional scanning had 450 EPI volumes, and the first five volumes were discarded to allow for T1 equilibration effects. High-resolution anatomical images were acquired using a standard T1-weighted 3D MP-RAGE sequence. The voxel size of the T1 image was 1 × 1 × 1 mm3.

Data were pre-processed with Statistical Parametric Mapping software SPM8 (Wellcome Department of Imaging Neuroscience, London, http://www.fil.ion.ucl.ac.uk). Images were realigned to the first volume to correct for inter-scan head movements. Then, the mean EPI image of each subject was computed and spatially normalized to the MNI single subject template using the “unified segmentation” function in SPM8. This algorithm is based on a probabilistic framework that enables image registration, tissue classification, and bias correction to be combined within the same generative model. The resulting parameters of a discrete cosine transform, which define the deformation field necessary to move individual data into the space of the MNI tissue probability maps, were then combined with the deformation field transforming between the latter and the MNI single subject template. The ensuing deformation was subsequently applied to individual EPI volumes. All images were thus transformed into standard MNI space and re-sampled to 2 × 2 × 2 mm3 voxel size. The data were then smoothed with a Gaussian kernel of 8 mm full-width half-maximum to accommodate inter-subject anatomical variability.

#### Statistical analysis of imaging data

Data were analyzed employing a general linear model as implemented in SPM8. At the first level, the general linear model (GLM) was used to construct a multiple regression design matrix that included 2 conditions: “Near” and “Far”. Each condition was modeled by a boxcar reference vector (16.5 s) convolved with a canonical synthetic hemodynamic response function (HRF). Additionally, all instructions and the six head movement parameters derived from the realignment procedure were included as covariates of no interest. Task blocks, in which participants did not adhere to the task-instructions (error rates within such blocks were above 60%), were separately modeled as another regressor of no interest. This occurred in 4 participants, who each misperformed in 1 block (the entire experiment consisted of 48 blocks). Data were highpass-filtered at 1/128 Hz. Parameter estimates were subsequently calculated for each voxel using weighted least squares to provide maximum likelihood estimators based on the temporal autocorrelation of the data. No global scaling was applied. For each subject, the simple main effect was calculated for the near and far conditions, respectively. The two first-level individual contrast images were then fed into a 1 × 2 within-participants ANOVA at the second group level employing a random-effects model (flexible factorial design in SPM8 including an additional factor modeling the subject means). In the modeling of variance components, we allowed for violations of sphericity by modeling non-independence across parameter estimates from the same subject and allowing unequal variances both between conditions and participants using the standard implementation in SPM8. We were interested in the differences between the “Near” vs. “Far” conditions. Areas of activation were identified as significant only if they passed a conservative threshold of *p* < 0.001, corrected for multiple comparisons (family-wise error correction, FWE) at the cluster level, with an underlying voxel level of *p* < 0.001, uncorrected^50^.

#### Psychophysiological interaction (PPI) analysis

In order to further investigate how the occipital visual cortex was modulated by the higher-order cortical areas to generate size constancy in the virtual 3D environment, we used bilateral IOG as a source region (derived from the conjunction analysis between the main effect of the depth of objects in the 3D experiment, i.e. “Far > Near”, and the main effect of the physical size of stimuli in the 2D experiment, i.e. “Small > Large”) to estimate the context-specific functional modulation of neural activity across the brain using psychophysiological interaction (PPI) analysis. PPI analysis allows for detecting regionally specific responses in one brain area in terms of the interaction between inputs from another brain region and a cognitive/sensory process^50^. We used the contrast “Far > Near” as the psychological factor, and used the neural activity in the left and right IOG as the physiological factor, respectively. For each subject, the “Far > Near” contrast was first calculated at the individual level. Subsequently, each subject’s individual peak voxel was determined as the maximally activated voxel within a sphere of 16mm radius (i.e., twice the smoothing kernel) around the coordinates of the peak voxel within bilateral IOG (Fig. 1c, upper panel and Table 1e) from the second level group analysis. Individual peak voxels from every subject were located in the same anatomical structure (left IOG: x = −29 ± 6, y = −93 ± 4, z = −7 ± 5, right IOG: x = 31 ± 5, y = −90 ± 5, z = −6 ± 6). Next, the time series of these regions were extracted from a sphere of 4 mm radius (twice the voxel size) around the individual peak voxels (without deconvolution because of the block design). PPI analysis at the individual level employed one regressor representing the extracted time series in the given region of interest in bilateral middle occipital gyrus (the physiological variable), one regressor representing the psychological variable of interest, that is “Far > Near”, and a third regressor representing the cross product of the previous two (the psychophysiological interaction term). An SPM was calculated to reveal areas whose activation was predicted by the psychophysiological interaction term, with the physiological and the psychological regressor being treated as confound variables. The PPI analysis was carried out for each subject, and then entered into a random-effects group analysis (*p* < 0.001, FWE corrected for multiple comparisons at cluster level with an underlying voxel threshold at p < 0.005, uncorrected).

The bilateral SOG (derived from the conjunction analysis between “3D_Near > 3_Far” and “2D_Large > 2D_Small”, Fig. 1C, lower panel) were also used as the seeds to perform the PPI analysis. We used the contrast “Near > Far” as the psychological factor, and used the neural activity in the left and right SOG as the physiological factor, respectively. Individual peak voxels from every subject were located in the same anatomical structure (left SOG: x = −16 ± 7, y = −89 ± 4, z = 26 ± 7, right SOG: x = 20 ± 8, y = −85 ± 3, z = 34 ± 5). All the other procedure was the same as above.

### 2D Experiment

#### Participants

Twenty five healthy volunteers (14 male and 11 female, 22 ± 3 years old) participated in the 2D control experiment (none of them participated in the virtual 3D experiment). They were all right-handed and had normal or corrected-to-normal visual acuity. None of them had a history of neurological or psychiatric disorders. Written and informed consent was obtained from all subjects before participation according to the guidelines of South China Normal University’s Human Subjects Review Board. All experimental protocols were approved by the Academic Committee of School of Psychology, South China Normal University. All methods were carried out in accordance with approved guidelines and regulations. All the participants were paid for their participation.

#### Experimental design and stimuli

The experimental design was the same as that in the virtual 3D experiment, except that all the stimuli were presented on a 2D screen (Fig. 5b): the retinal sizes/images and the retinal positions of the objects were identical to those in the virtual 3D experiment, but the depth dimension was removed. Thus, the two “Large” and “Small” conditions in the 2D experiment corresponded to the “Near” and “Far” conditions in the 3D experiment. Due to the mal-functioning of the response pad during one scanning day of the 2D experiment (responses on more than half of the trials were not recorded), we were not able to collect the online behavioural responses of six participants. Based on the behavioral data from our previous pilots and from the practice session prior to the MR scanning, however, we are sure that the six participants in our 2D control experiment are able to perform the present behavioral tasks very well. Therefore, neural data of the six participants are not excluded from the fMRI analysis.

#### Data acquisition, pre-processing and statistical analysis of imaging data

All of the settings were the same as those in the virtual 3D experiment.

#### Psychophysiological interaction (PPI) analysis

The same as those in the virtual 3D experiment. Bilateral IOG (Fig. 1c upper panel and Table 1e) and bilateral SOG (Fig. 1c lower panel and Table 1f) were used as the physiological factor (source region), respectively, and the contrast “Small > Large” and “Large > Small” as the psychological factor, respectively. Individual peak voxels from every subject were located in the same anatomical structure (left IOG: x = −30 ± 4, y = −95 ± 3, z = −11 ± 5, right IOG: x = 32 ± 5, y = −93 ± 4, z = −7 ± 5, left SOG: x = −12 ± 4, y = −92 ± 6, z = 15 ± 7, right SOG: x = 12 ± 3, y = −90 ± 5, z = 21 ± 6).

### The psychophysics control experiment

It has been suggested that there exist differences between depth perception in the real and the virtual 3D environment. For example, compared to the real 3D world, distances from the observer were underestimated in the virtual environments^22^. Furthermore, distance miscalculation was universal in various virtual reality devices^19–21^. Therefore, to further test whether human subjects perceive the far objects as the same objects as the near objects in the present virtual 3D environment, we conducted another psychophysics control experiment.

#### Participants

Nine healthy volunteers (6 female; 24 ± 1.5 years old) participated in the present experiment. They were all right-handed and had normal color vision and visual acuity. None of them had a history of neurological or psychiatric disorders. Written consent was obtained from all subjects before participation according to the guidelines of South China Normal University’s Human Subjects Review Board. They were paid for their participation.

#### Experimental design and stimuli

The 3D stimuli and their spatial locations in the near and far space were exactly the same as those in the virtual 3D fMRI experiment. At the start of each trial, the 3D_Near objects were firstly presented as the reference objects until participants pressed the middle button of the mouse. The 3D_Far objects of a random initial size were then presented. Also, the far space stimuli were presented at the same height (x = 0, and y = 0, at the level of the eyes) as the near space stimuli, and tilted by 13° toward the participants.

Participants were instructed to modulate the size of the far objects until they perceived the far objects as the same objects as the near objects. The size of the objects became 2% larger if the participants pressed the left button of the mouse, and 2% smaller if the participants pressed the right button of the mouse. The correspondence between the two buttons on the mouse and the increasing/decreasing size of the far objects was counter-balanced across participants. When the participants finished the adjustment on each trial, they were instructed to press the middle button of the mouse to go to the next trial. There were 20 trials for each participant.

#### Statistical analysis of psychophysics data

The mean modulated the size of the far plate was submitted to a one sample *t* test to compare with the real size of the far plate (7.6° in visual angle) in the virtual 3D fMRI experiment. The results suggested that there was no statistically significant difference between the modulated size (7.77° ± 0.19° of visual angle) and the real size of the far plate (7.67° in visual angle), *t* (8) = 0.345, *p* > 0.05, Cohen’s *d* = 0.244. These results thus indicated that participants perceived the 3D_Far object as the same object as the 3D_Near object in the virtual 3D experiment, irrespective of whether the participants were asked to subjectively adjust the size of the far objects or the near objects were directly put in the far space.
